# The complex biology of aryl hydrocarbon receptor activation in cancer and beyond

**DOI:** 10.1016/j.bcp.2023.115798

**Published:** 2023-10

**Authors:** Christiane A. Opitz, Pauline Holfelder, Mirja Tamara Prentzell, Saskia Trump

**Affiliations:** a German Cancer Research Center (DKFZ), Heidelberg, Division of Metabolic Crosstalk in Cancer and the German Cancer Consortium (DKTK), DKFZ Core Center Heidelberg, 69120 Heidelberg, Germany; b Neurology Clinic and National Center for Tumor Diseases, 69120 Heidelberg, Germany; c Faculty of Bioscience, Heidelberg University, 69120 Heidelberg, Germany; d Molecular Epidemiology Unit, Berlin Institute of Health at Charité and the German Cancer Consortium (DKTK), Partner Site Berlin, a partnership between DKFZ and Charité –Universitätsmedizin Berlin, 10117 Berlin, Germany

**Keywords:** AHR activation, Ligand diversity, AHR expression, ARNT, AHRR, Interaction with transcription factors, Crosstalk with signaling pathways, Non-genomic AHR effects, Epigenetic regulation, Posttranslational modifications, Degradation of AHR ligands, DNA methylation

## Abstract

The aryl hydrocarbon receptor (AHR) signaling pathway is a complex regulatory network that plays a critical role in various biological processes, including cellular metabolism, development, and immune responses. The complexity of AHR signaling arises from multiple factors, including the diverse ligands that activate the receptor, the expression level of AHR itself, and its interaction with the AHR nuclear translocator (ARNT). Additionally, the AHR crosstalks with the AHR repressor (AHRR) or other transcription factors and signaling pathways and it can also mediate non-genomic effects. Finally, posttranslational modifications of the AHR and its interaction partners, epigenetic regulation of AHR and its target genes, as well as AHR-mediated induction of enzymes that degrade AHR-activating ligands may contribute to the context-specificity of AHR activation. Understanding the complexity of AHR signaling is crucial for deciphering its physiological and pathological roles and developing therapeutic strategies targeting this pathway. Ongoing research continues to unravel the intricacies of AHR signaling, shedding light on the regulatory mechanisms controlling its diverse functions.

## Graphical abstract

The complex biology of AHR activation in cancer and beyond.

**Figure:**
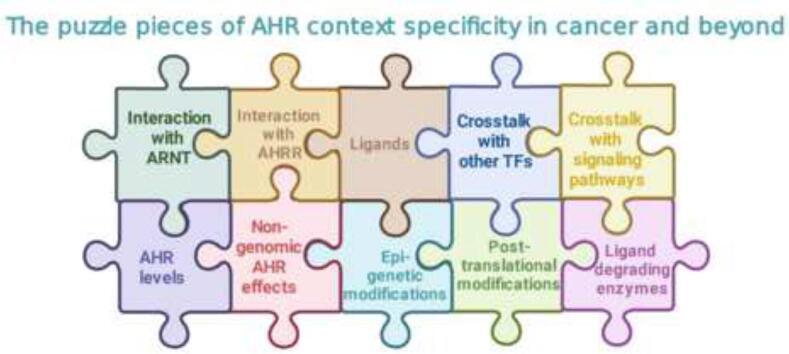
Graphical abstract depicts the contributing factors to AHR's context specificity. Created with BioRender.com (agreement number: HI25TPR0Z5)

## 1 Introduction

**Fig. 1:**
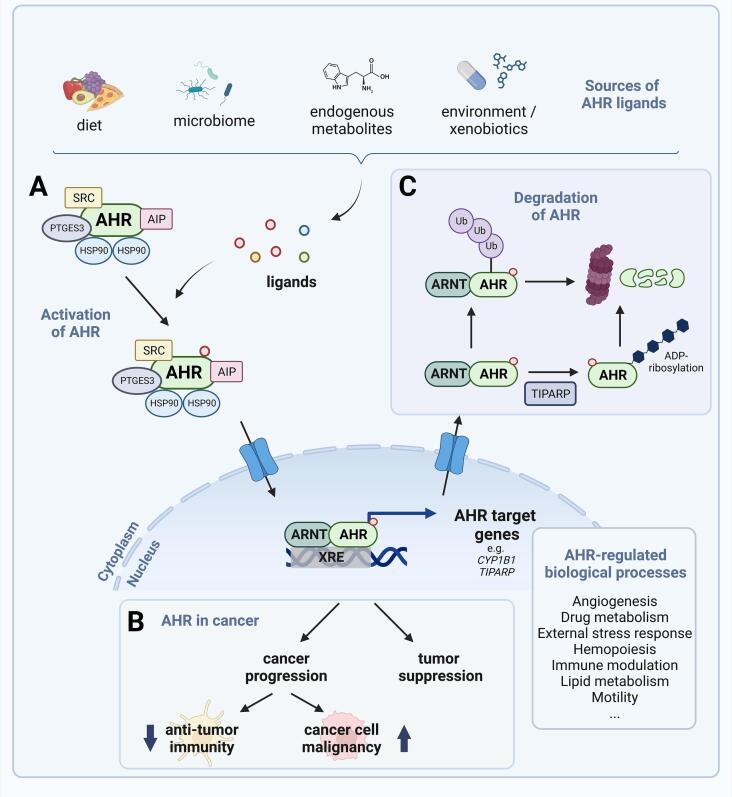
Schematic representation of canonical AHR signaling, AHR effects in cancer and AHR degradation. (A) *Canonical AHR signaling*: In its inactive state the AHR resides in the cytoplasm and forms a complex with 90 kDa heat shock proteins (HSP90), the AHR-interacting protein (AIP), the co-chaperone PTGES3 (prostaglandin E synthase 3, p23) and the protein kinase SRC. AHR ligands from different sources activate the AHR, which leads to its nuclear translocation. In the nucleus, AHR heterodimerizes with the AHR nuclear translocator (ARNT) and induces the transcription of its target genes by binding to xenobiotic response elements (XREs) in their promoter sequences. The induction of AHR target genes is highly cell type and tissue specific. Different AHR target genes mediate diverse biological functions that result in different outcomes. (B) *AHR in cancer*: AHR activation can promote tumor progression by inhibiting anti-tumor immunity and enhancing the malignancy of cancer cells, but it can also exert tumor suppressive functions. These divergent effects of AHR in cancer likely stem from the complexity of its activation and functions, which are cell type-, ligand- and context-specific. (C) *Degradation of AHR*: After transcriptional activation, AHR is exported from the nucleus to the cytoplasm where it is degraded by the proteasome in a ubiquitin-dependent manner. AHR degradation requires ligand binding and the presence of ARNT. ADP-ribosylation of AHR by the AHR target gene TCDD-inducible poly (ADP-ribose) polymerase (TIPARP) also increases the susceptibility of AHR to degradation. Taken together, AHR degradation ensures limitation of AHR-mediated gene regulation. Created with Biorender.com

In addition to its many other functions, the ligand-activated transcription factor aryl hydrocarbon receptor (AHR) is an important regulator of tumor progression. By modulating both tumor cell intrinsic malignant properties as well as anti-tumor immunity, AHR can promote the progression of tumors. However, depending on the tumor type and stage of tumor development, it can also exert tumor suppression (Fig. 1).

The AHR is a member of the basic helix-loop-helix (bHLH), and periodic circadian protein (PER) – AHR nuclear translocator (ARNT) – single minded protein (SIM) [PAS] superfamily of transcription factors that sense and respond to a plethora of different signals [1]. While other members of this family including the hypoxia inducible factors (HIFs) and the circadian locomotor output cycles kaput (CLOCK) sense alterations in oxygen tension or circadian rhythms, respectively, the AHR is the only bHLH-PAS transcription factor that is activated by small molecules.

In its inactive state the AHR resides in the cytoplasm and forms a complex with two 90 kDa heat shock proteins (HSP90), the AHR-interacting protein (AIP also known as XAP2 or ARA9), the co-chaperone prostaglandin E synthase 3 (PTGES3 also known as p23) and the protein kinase SRC proto-oncogene, non-receptor tyrosine kinase (SRC) [2]. AHR contains a nuclear localization sequence (NLS) that is necessary for translocation from the cytoplasm to the nucleus [3], [4], [5]. Ligand binding exposes the NLS enabling AHR translocation. In the nucleus, the AHR binds to the AHR nuclear translocator (ARNT, also known as HIF1β) and functions as a transcription factor through binding to xenobiotic response elements (XREs) of its target genes. Binding of the AHR-ARNT heterodimer to XREs is considered as canonical AHR signaling (Fig. 1A).

The AHR was originally discovered to mediate the toxic effects of environmental pollutants such as 2,3,7,8-tetrachlorodibenzo-*p*-dioxin (TCDD) [1]. Adverse health effects related to TCDD exposure were first illustrated as a consequence of accidental releases from chemical plants, such as in 1949 in Nitro, West Virginia and in 1976 in Seveso, Italy [6]. Exposure to Agent Orange during the Vietnam War has also been linked to an increase in cancer incidence and the occurrence of birth defects in offspring, primarily attributed to TCDD as one of the components of the defoliant [7], [8].

In the past decades, the physiological role of the AHR in regulating important biological functions including angiogenesis [9], [10], drug metabolism [11], hematopoiesis [12], cell motility [13] and immunity [12], [14], [15] has been firmly established (Fig. 1A). It has become clear that AHR activation plays important distinct roles in cancer biology even in the absence of environmental toxins [16].

The AHR is a key regulator of tumor progression by modulating both tumor cell intrinsic malignant properties as well as anti-tumor immunity (Fig. 1B). Many tumors express high levels of the AHR and produce endogenous AHR agonists, thus taking advantage of AHR activation to promote tumor cell intrinsic malignant properties and to suppress anti-tumor immune responses [14], [17], [18]. Specifically, the AHR drives cancer cell migration, invasion, and survival, regulates cell cycle progression and promotes cancer stem cell characteristics [14], [19], [20], [21], [22]. Simultaneously, it inhibits anti-tumor immunity by recruiting immunosuppressive tumor associated-macrophages [23], suppressing T cell proliferation, and inducing T cell exhaustion and death [15], [24], [25], [26]. These results have prompted the development of drugs inhibiting the AHR [27], [28] or upstream AHR agonist-producing enzymes, albeit until now with limited success [29], [30], [31], [32]. Resistance to pharmacological inhibitors of upstream AHR agonist-producing enzymes could be due to compensation through other AHR ligand-producing pathways. Further, cell type- or ligand-specific differences in the efficacy of AHR inhibition might hamper strategies that target the AHR directly. Moreover, differences between the human and mouse AHR, which show species-specific ligand selectivity as well as different transcriptional responses to a given ligand [33], limit the *in vivo* investigation of AHR functions. Various clinically approved drugs including omeprazole, 4-hydroxytamoxifen, flutamide, leflunomide, and nimodipine, activate the AHR without increasing cancer risk [34], [35]. This underscores that AHR activation does not necessarily promote cancer. Moreover, the AHR also exerts tumor-suppressive effects (Fig. 1B) and has been shown to inhibit tumor formation [36], [37] and metastasis [38], [39], [40]. These divergent effects of the AHR in cancer likely stem from the complexity of its activation and effects, which are cell type-, ligand- and context-specific but are poorly defined. In this review, we will provide a general overview about the complex biology of AHR activation and not limit ourselves to cancer, because the effects of multiple mechanisms that modulate AHR activity have not yet been explored in the cancer context. However, to exploit the AHR for cancer therapy a comprehensive understanding of its complex activation and diverse biological outcomes is necessary and we expect the described mechanisms to also apply to cancer.

## 2 The diversity of ligand-dependent AHR responses

While it was initially believed that only planar molecules with hydrophobic structures activate the AHR, it is now recognized that a plethora of structurally diverse environmental and endogenous ligands can activate this receptor [19], [41], [42]. Various natural compounds modulate AHR activity. Tryptophan (Trp) catabolites [43], [44], [45], [46], eicosanoids [47], bilirubin [48], and cAMP [49] exert agonistic effects, while vitamin B12 and folic acid have been reported to be AHR antagonists [50].

Binding and activation of the AHR by diverse compounds results in the induction of diverse genes and mediates a variety of effects. One example for this phenomenon is that the high affinity AHR agonists 6-formylindolo[3,2–*b*]carbazole (FICZ) and TCDD exerted opposing effects on CD4^+^ T cell differentiation [51], [52]. A study following up on this observation optimized the dose and timing of administration of FICZ for TCDD-equivalent cytochrome P450 family 1 subfamily A member 1 (*Cyp1a1*), AHR target gene, induction and observed the same dynamics and responses for TCDD and FICZ [53]. This finding suggests that the dose and the duration of AHR activation by high-affinity AHR ligands determine its effects. However, also binding to different sites of the AHR may contribute to the ligand-specificity of AHR activation. The BRAF inhibitor vemurafenib binds the AHR as it displaces radiolabeled TCDD, it leads to nuclear translocation of the AHR, but not to AHR-ARNT binding as evidenced by proximity ligation assay. In line, vemurafenib reduces XRE-luciferase activity and induces neither the mRNA nor the activity of CYP1A1. Vemurafenib and TCDD induce specific and mutually exclusive transcriptome signatures. TCDD or the AHR antagonist CH-223191 prevent vemurafenib-mediated effects. The difference between TCDD/CH-223191 and vemurafenib was attributed to the fact that they bind to different positions of the AHR that are close enough to each other to hinder each other’s binding [54]. This finding suggests that different binding pockets of AHR may exert different effects and also influence canonical AHR activation.

Moreover, overlap of ligands for AHR and other promiscuous receptors may affect the heterogeneity of responses to different ligands. Denison and colleagues found a striking overlap between agonists of AHR and the nuclear receptor pregnane X receptor (PXR), suggesting that activation of AHR and crosstalk with other transcription factors may contribute to ligand-specific AHR responses [55].

Taken together, there is a high degree of diversity in ligand-dependent AHR responses and multiple excellent reviews have recently covered this topic, to which we direct our readers for a detailed overview [55], [56], [57], [58].

Tumors express different enzymes that generate AHR ligands and their differential expression may contribute to the context-specificity of AHR activation as well as resistance to specific inhibitors. Derivatives of the essential amino acid Trp represent an important class of endogenous AHR ligands [19], [20], [33]. The kynurenine (Kyn) pathway, initiated by indoleamine-2,3-dioxygenase 1/2 (IDO1/2) [59] or tryptophan-2,3-dioxgenase (TDO2) [43], [44], [60], [61], [62], generates the AHR agonists Kyn and kynurenic acid (KynA) [24], [43], [63], [64]. The L-amino acid oxidase interleukin-4-induced-1 (IL4I1) degrades Trp to indole-3-pyruvic acid (I3P) that yields the AHR agonists indole-3-aldehyde and kynurenic acid [45]. In healthy tissues, high *IDO1* mRNA expression is present in lung, while *TDO2* and *IL4I1* show high expression in liver and testis, respectively [45]. Nearly all tumors express elevated levels of at least one of the Trp-degrading enzymes and depending on the tumor type AHR activation associates with the expression of one or more of these enzymes [45]. However, several tumors exist that show high AHR activity without association to a Trp-degrading enzyme, suggesting that other ligands could be responsible for AHR activation.

Apart from endogenous metabolites, exposure to AHR ligands from the diet, the microbiome, as well as environmental toxicants including tobacco smoke, particulate matter or exposure to factors triggering the generation of AHR agonists such as UVB light also plays an important role for AHR activation in tumor development and progression [56], [65].

Cryo-EM structures of indirubin bound to the human AHR-HSP90-AIP complex and the unliganded mouse AHR-HSP90-PTGES3 complex with or without AIP have recently become available [66], [67]. The ligand binding pocket comprises a primary and secondary binding site. Upon binding to indirubin, only the primary site is occupied. In this primary binding site, specific polar amino acid residues determine the selectivity for planar hydrophobic molecules with an electron-rich π-system to form hydrogen bonds and π-π interactions. Furthermore, the cryo-EM structure indicates that the secondary binding site appears to be less geometrically constrained, thereby allowing larger compounds to fit [66]. The structure of the AHR PAS-B domain provides the basis for a better understanding of ligand-AHR interactions.

## 3 AHR expression

The AHR is ubiquitously expressed although its expression levels vary between tissues and cell types [68]. In human tissues, the AHR is expressed particularly in the lung, liver, urinary bladder, bone marrow, and highest in the placenta. AHR expression has been reported to be regulated by glucocorticoid signaling at concentrations that activate the glucocorticoid receptor (GR). Dexamethasone (DEX) increased AHR mRNA, protein, and TCDD-binding by approximately 50% in Hepa-1 mouse hepatoma cells, which was blocked by a GR antagonist [69]. In contrast, DEX reduced AHR mRNA and protein in human hepatocytes and led to accelerated AHR degradation in the human choriocarcinoma cell line JEG-3 [70], [71]. Taken together, these findings suggest that the effects of DEX on AHR expression differ between mice and humans.

Recently, deprivation of Trp was discovered to induce AHR mRNA and protein levels resulting in enhanced AHR activity. AHR induction in response to Trp limitation is mediated through potentially cell type-specific mechanisms involving nuclear factor erythroid 2-related factor 2 (NRF2) signaling in HEK 293 cells and epidermal growth factor receptor (EGFR)-rat sarcoma (RAS)-signaling to the mammalian target of rapamycin complex 1 (MTORC1) and p38/mitogen activated protein kinase (MAPK) in glioblastoma cells [64], [72].

## 4 AHR-ARNT interaction

Binding of ligands leads to a conformational change of the AHR complex in the cytoplasm and subsequent nuclear translocation of the AHR. In the nucleus, the AHR forms a heterodimer with ARNT (aka HIF1β) and binds to specific XREs with a consensus sequence (5ʹ-TNGCGTG-3ʹ) to regulate multiple target genes [73].

ARNT is ubiquitously expressed. However, ARNT protein levels are influenced by hypoxia and hypoxia mimetics such as cobalt(II)-chloride and dimethyloxalylglycine (DMOG) in a cell line-specific manner, indicating that cell type and oxygen availability may impact AHR activation by modulating ARNT levels [74].

In mice, the ARNT paralog ARNT2, which is ∼ 80% identical to ARNT in the N-terminal bHLH and PAS regions, but is more divergent in the C terminus, is primarily expressed in the kidney, central nervous system, and retinal epithelium [75], [76]. In murine Hepa-1 hepatoma cells it was shown that ARNT and ARNT2 dimerize equally with the AHR in the presence of TCDD and that ARNT2 outcompetes ARNT for binding to the AHR when expressed in excess. However, activation of the AHR with 3-methylcholanthrene or benzo[*a*]pyrene (BaP) results in predominant formation of AHR-ARNT complexes. ARNT2 expression in cells with reduced ARNT protein results only in minimal induction of CYP1A1 protein. However, the expression of ARNT2 reduces TCDD-mediated induction of endogenous CYP1A1 protein by 30%, even though AHR-ARNT2 complexes could not be detected in nuclear extracts. Taken together, ARNT2 has the ability to dimerize with the liganded AHR *in vitro* but it may rather inhibit than enhance canonical AHR activation [75]. Further studies are necessary to understand the role of ARNT2 in AHR signaling.

ARNT itself is present in a long and a short splice isoform, ARNT isoform 1 and ARNT isoform 3, respectively [77], [78], [79]. The two isoforms differ by only 15 amino acids. In naïve B and T cells the two isoforms are present at equal ratios, whereas lymphoid malignancies show an increase in the levels of ARNT isoform 1, which promotes their growth and survival [79]. The RNA-binding protein fox-1 homolog 2 (RBFOX2) was upregulated in lymphoid malignancies, which enhanced ARNT isoform 1 levels [78]. The ratio between ARNT isoform 1 and ARNT isoform 3 regulates AHR target gene expression. Suppression of ARNT isoform 1 enhances AHR responsiveness to ligand activation and mediates inflammation, while suppression of ARNT isoform 3 reduces AHR responsiveness to ligand activation and mediates immunosuppression.

## 5 Degradation of AHR

After transcriptional activation, the AHR is exported from the nucleus to the cytoplasm where it is subject to ubiquitin-dependent proteasomal degradation [80] (Fig. 1C). AHR degradation requires ligand binding and the presence of ARNT [81]. Recently, ubiquitin carboxyl terminal hydrolase L3 (UCHL3) was demonstrated to interact with, deubiquitylate, and stabilize AHR, thereby promoting the stem-like characteristics of non-small cell lung cancer cells [82]. The consequence of blocking AHR degradation in cell culture appears to be an increase in both the magnitude and duration of gene regulation by the AHR-ARNT complex. Thus, the physiological role of AHR degradation may be to limit AHR-mediated gene regulation [83].

Of note, AHR degradation is also regulated by the AHR target gene TCDD-inducible poly (ADP-ribose) polymerase (TIPARP), which ADP-ribosylates AHR, hence increasing its susceptibility to degradation [83], [84], [85], [86], [87]. Taken together, also the dynamics of AHR degradation may contribute to the context-specificity of AHR activation.

## 6 Interaction with AHRR

The AHR repressor (AHRR) is induced by binding of the AHR to an XRE in its promoter sequence and exerts AHR inhibition [88]. It also is a bHLH/PAS protein and strongly resembles the AHR in its N-terminal half consisting of the DNA-binding bHLH and the PAS-A domain, but it lacks the C-terminal ligand-binding PAS-B domain and the glutamine-rich transactivation domain. The mechanisms of AHR inhibition mediated through AHRR appear to be manifold including competition for ARNT- and XRE-binding [88], [89], closing of chromatin by recruiting co-repressors to the XREs of AHR target genes [90] as well as mechanisms independent of competition for ARNT or XRE-binding [91]. In humans, AHRR expression is highest in the testis (https://gtexportal.org/home). In addition to AHR, nuclear factor kappa-light-chain-enhancer of activated B cells (NF-κB) and specificity protein (SP) transcription factors SP1 and SP3 may regulate AHRR expression, possibly contributing to the context-specificity of AHR activation [92].

Using an AHRR reporter and knockout mouse strain, Brandstätter and colleagues found that AHRR expression in naïve mice is very cell type-specific [93]. AHRR expression is mainly restricted to immune cells and is most prominent in the cutaneous and intestinal barrier. The highest frequency of AHRR expressing cells is present in the small intestine, including CD11c^+^ myeloid cells, T cells, type 3 innate lymphoid cells and intraepithelial lymphocytes, suggesting that AHRR may restrain excessive AHR activation in immune cells of barrier organs. In contrast, AHRR is barely expressed in liver and undetectable in intestinal epithelial cells. In LPS-induced septic shock, Ahrr knockout confers enhanced resistance, which is opposite to the high LPS sensitivity of Ahr-deficient mice. On the other hand, Ahrr knockout exacerbates colitis similar to Ahr deficiency [93]. This discrepancy may be due to the restricted tissue- and cell type-specific expression of AHRR, which is different from the ubiquitous expression of the AHR.

While AHRR DNA hypomethylation is associated with increased lung cancer risk, AHRR is silenced by DNA hypermethylation in multiple human malignancies including breast, cervix, colon, lung, ovary and stomach, suggesting that it may exert tumor suppressive properties in these tumor entities [94]. Taken together AHRR expression is modulated by environmental exposures as well as the cell types it is expressed in, hence possibly contributing to the context-specificity of AHR activation.

## 7 Interaction with other transcription factors and crosstalk with other signaling pathways

**Fig. 2:**
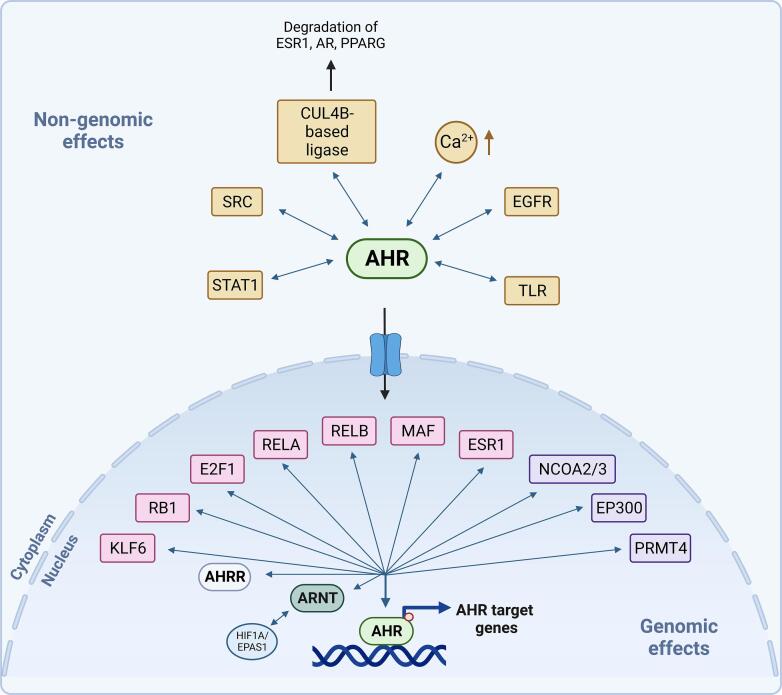
Non-genomic and genomic effects of AHR. *Non-genomic effects of AHR*: In the cytoplasm, AHR interacts with the non-receptor tyrosine kinase SRC proto-oncogene as well as with signal transducer and activator of transcription 1 (STAT1) and modulates their signaling. AHR also crosstalks with epidermal growth factor receptor (EGFR) and Toll-like receptor (TLR) signaling and regulates intracellular calcium levels and calcium-dependent signaling pathways. AHR is involved in the assembly of a Cullin 4B (CUL4B)-based ubiquitin ligase complex, thereby promoting the proteasomal degradation of estrogen receptor alpha (ESR1), androgen receptor (AR) as well as peroxisome proliferator-activated receptor gamma (PPARG). *Genomic effects of AHR*: In canonical AHR signaling, AHR forms a heterodimer with AHR nuclear translocator (ARNT). The hypoxia inducible factors HIF1A and EPAS1 (endothelial PAS domain protein 1, HIF2α) also bind to ARNT and compete with ligand-bound AHR for the interaction with ARNT, thereby reducing AHR activity. The AHR target gene AHR repressor (AHRR) inhibits AHR signaling through mechanisms including competition for ARNT- and XRE-binding, closing of chromatin by recruiting co-repressors to the XREs of AHR target genes, as well as mechanisms independent of competition for ARNT or XRE-binding. Besides heterodimerizing with ARNT, AHR can interact with multiple transcription factors (pink) including KLF6 (KLF transcription factor 6), RB1 (retinoblastoma transcriptional corepressor 1), E2F1 (E2F transcription factor 1) [97], [98], [99], the NF-κB subunits RELA (RELA proto-oncogene) and RELB (RELB proto-oncogene) [104], [105], MAF (MAF BZIP transcription factor), and estrogen receptor alpha (ESR1). AHR also interacts with numerous co-regulators (purple) such as the nuclear receptor coactivators 2 and 3 (NCOA2/3), E1A binding protein P300 (EP300) and protein methyltransferase 4 (PRMT4). Created with Biorender.com. (For interpretation of the references to colour in this figure legend, the reader is referred to the web version of this article.)

The non-canonical interaction of the AHR with other transcription factors and co-regulators as well as its crosstalk with other signaling pathways also contribute to the context-specificity of AHR activation [95] (Fig. 2).

The AHR binds to many different transcription factors such as KLF transcription factor 6 (Krueppel-like factor 6) [96], RB1 (retinoblastoma transcriptional corepressor 1), E2F1 (E2F transcription factor 1) [97], [98], [99], the NF-κB subunits RELA (RELA proto-oncogene) [100], [101], [102], [103] and RELB (RELB proto-oncogene) [104], [105], MAF (MAF BZIP transcription factor), and estrogen receptor alpha (ESR1) [106], [107], [108], [109], [110], [111], [112], [113], [114]. As ESR1, for instance, is highly expressed in endometrium, vagina, cervix, fallopian tube and breast, it affects AHR activation in a tissue- and sex-specific manner. AHR binding to RB1 represents another example of context-specificity as AHR only binds to hypophosphorylated RB1 [115], [116], which is only present in G0 and G1 phase and hence can only exert effects in specific phases of the cell cycle.

Furthermore, HIF1α and HIF2α also bind to ARNT and compete with ligand-bound AHR for the interaction with ARNT, hence reducing AHR activity [20], [21]. Changes in oxygen tension may therefore also contribute to the context-specificity of AHR activation.

AHR crosstalk with other transcription factor and signaling pathways has extensively been reviewed and we therefore direct our readers to excellent reviews that have comprehensively covered the landscape of AHR regulators and co-regulators [95] as well as AHR crosstalk with other signaling pathways [117], [118], including EGFR, signal transducer and activator of transcription (STAT) as well as Toll-like receptor (TLR) and NF-κB signaling.

The direction of the transcriptional response, i.e. repression or activation, is not only determined by the transcription factors themselves, but also by their recruitment of co-activators or co-repressors. AHR has been described to interact with several of these co-regulators, e.g. nuclear receptor coactivators 2 and 3 (NCOA2/3), E1A binding protein P300 (EP300), or protein methyltransferase 4 (PRMT4), which has recently been extensively reviewed by Gargaro and colleagues [95].

## 8 Non-genomic effects of AHR

Emerging research has uncovered non-genomic effects of AHR that extend beyond its role in gene regulation (Fig. 2).

In the cytoplasm, AHR interacts with the non-receptor tyrosine kinase, SRC [119]. SRC acts as an proto-oncogene in a plethora of cancer entities [120]. Activation of AHR by different ligands leads to the phosphorylation of SRC and subsequent activation of downstream proteins, such as the EGFR and focal adhesion kinase (FAK) [121], [122], [123]. Of note, the AHR/SRC axis has recently been discovered as a new therapeutic vulnerability that triggers resistance to BRAF (B-Raf proto-oncogene, serine/threonine kinase) inhibitors in melanoma [123]. Furthermore, inhibition of both AHR and SRC synergistically reduced androgen receptor (AR) signaling and the growth of prostate cancer cells [124].

Moreover, cytoplasmic AHR also interacts with STAT1. Activation of JAK-STAT signaling leads to release of phosphorylated STAT1 to the cytoplasm, where it can form a heterodimer with AHR that is thought to repress formation and nuclear translocation of STAT dimers [117], [125].

Also non-transcriptional effects of AHR activation show ligand dependency as alterations in multiple post-transcriptional modifications (PTMs), which were dependent on the administered AHR ligand, were detected by mass spectrometry two hours after stimulation of monocyte-derived macrophages with BaP or FICZ in the presence or absence of lipopolysaccharide (LPS) [126]. Both BaP and FICZ resulted in ubiquitination of Rac Family Small GTPase 1 (RAC1) [126]. However, although the concentrations of BaP and FICZ led to comparable upregulation of *CYP1B1* and *AHRR* mRNA, ubiquitination and phosphorylation was ∼ 5-fold more regulated in response to BaP than to FICZ [126].

Moreover, AHR mediates non-transcriptional actions through the assembly of a CUL4B-based ubiquitin ligase complex. Through this mechanism AHR promotes the ligand-dependent proteasomal degradation of specific substrates including ESR1, AR as well as peroxisome proliferator-activated receptor gamma (PPARG) [110], [127], [128].

Furthermore, AHR has been implicated in the regulation of intracellular calcium levels and calcium-dependent signaling pathways. Pyrene, for instance, was reported to act as an antagonist of AHR transcriptomic responses, but it induced an AHR-dependent increase in calcium within minutes after exposure [129].

Taken together the diverse non-genomic effects of AHR may also contribute to the context-specificity of AHR activation.

## 9 Different post-translational modifications may affect the AHR in different cell types

PTMs are also known to regulate a variety of proteins that help the cell adapt to environmental and metabolic changes, and thus can lead to context-specific responses [130]. PTMs can be either reversible chemical changes of amino acid residues (mainly serine (S), tyrosine, threonine, lysine (K)) or addition of polypeptides or proteins [131], [132]. While regulation of nuclear receptors by PTMs is well known, less is known about how PTMs control AHR activation. Although AHR is not a member of the nuclear receptor superfamily, the receptors share similar features, such as being cytosolic receptors that migrate to the nucleus upon ligand activation as well as possessing both a ligand-binding and a DNA-binding domain [133]. It hence is conceivable that PTMs may also play an important role in the context-specific regulation of AHR activation.

**Fig. 3:**
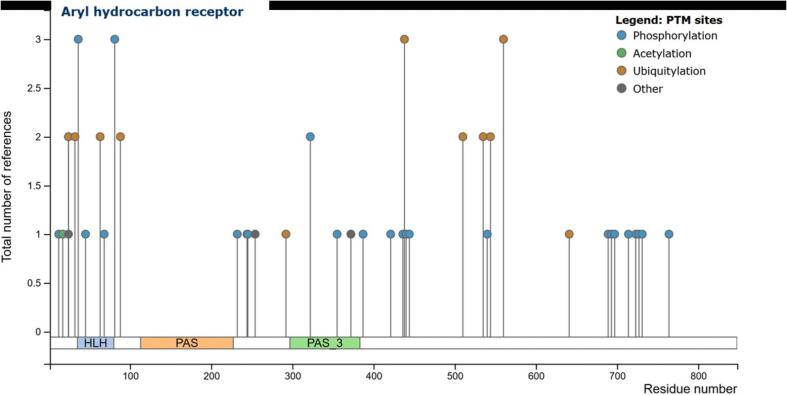
Reported posttranslational modifications of AHR. Depicted are posttranslational modifications (phosphorylation, acetylation, ubiquitination and others) at the specific residue number (x-axis) and the respective total number of references (y-axis) which includes records from high throughput papers (HTP, in which the modification site was assigned using only mass spectrometry) as well as from low throughput papers (LTP, in which this modification site was determined using methods other than mass spectrometry. Source: PhosphoSitePlus v6.7.1.1 (https://www.phosphosite.org/).

Indications that PTMs may be involved in ligand binding, nuclear import, DNA binding and degradation of the AHR have been accumulating for decades. With the development of high-throughput OMICs technologies, several additional PTMs have been discovered in recent years. An overview of PTMs of AHR identified by high-throughput methods and other low-throughput studies is shown in Fig. 3.

### 9.1 Phosphorylation

Cell- and context-specific expression and activity of kinases and phosphatases alter the phosphorylation status of transcription factors within the cell, thus regulating nuclear translocation, protein–protein interaction, and DNA binding [134], [135]. For AHR, phosphorylation was suggested to have effects on ligand binding, nuclear import, and DNA complex formation. Although Dolwick et al. identified several potential phosphorylation sites in the sequence of mouse and human AHR in the early 1990 s that are likely recognized by protein kinase C (PKC), cAMP-dependent protein kinase (PKA), and casein kinase II (CKII), relatively few studies to date have provided evidence for kinases that directly mediate phosphorylation of the AHR [136].

Modulation of ligand binding by phosphatases is established for various nuclear receptors [137], including the GR [138], [139] and the ESR1 [140] and a similar response was also assumed for TCDD binding to the AHR [141]. However, other studies showed that phosphorylation of AHR does not alter ligand binding of TCDD [139], [142].

Since AHR localization is a critical step in the induction of target genes, PTMs regulating AHR shuttling could also explain the cell type specificity of the AHR. For other shuttling proteins, phosphorylation and dephosphorylation have previously been shown to regulate their intracellular distribution [143]. For AHR, phosphorylation at PKC sites S12 or S36 adjacent to the bipartite basic amino acid segment of the NLS inhibits ligand-dependent nuclear translocation in monkey kidney fibroblast-like COS7 and cervical carcinoma HeLa cells [144]. In contrast, AHR phosphorylation at S36 by PKCθ enhances AHR nuclear import in T cells [145]. In addition, cell density, but not cell cycle, regulate AHR localization in human HaCaT keratinocytes through p38/MAPK-mediated phosphorylation of S68 in the NES, resulting in nuclear accumulation at low cell density [146]. These reports indicate that phosphorylation of AHR affects its localization differently in different cell lines and under different conditions. However, it is unknown whether phosphorylation of the AHR occurs before or after the ligand-induced release of the cytoplasmic co-chaperone complex.

Phosphorylation of AHR is also essential for its DNA binding and transcriptional activity. Early studies showed that phosphatases and kinase inhibitors suppressed DNA complex formation and TCDD-induced *CYP1A1* expression [142], [147], [148]. This is interesting, as phosphatase treatment of other transcription factors enhances DNA binding, likely due to removal of phosphate residues in the DNA binding domain that are electrostatically repelled from the phosphate backbone of the DNA [149]. This leads to the assumption that AHR is likely not phosphorylated in its bHLH DNA-binding domain. Chemical cleavage identified two regions in the C-terminal part of the AHR that are phosphorylated, one within or near a DNA-binding repressor domain (between amino acids 368 and 605) and one in the glutamine-rich C-terminal region (between amino acids 636–759) [142].

A growing body of evidence indicates the involvement of PKC in AHR phosphorylation. Some studies suggested PKC to regulate DNA binding and transcriptional activity of AHR [150], [151], [152]. However, this seems to be cell-type and context-specific as some authors have also reported conflicting results, showing that PKC activity modulation does not alter AHR nuclear translocation and XRE-binding in HepG2 and HeLa cells despite using similar PKC modulating compounds [153], [154]. Moreover, binding of AHR to DNA in response to TCDD was also detected in the absence of PKC activity in liver cytosol of guinea pigs, a species very sensitive to TCDD toxicity [155].

In addition to serine phosphorylation, phosphorylation on tyrosine residues is important in regulating AHR activity [156], [157] and inhibition of tyrosine kinases by genistein, abrogates the TCDD-induced *CYP1A1* induction, XRE reporter gene activity and DNA binding of the AHR in human keratinocytes [158].

Several mechanisms have been proposed for how phosphorylations may regulate DNA binding. While phosphorylation of AHR seems to be important for DNA binding, phosphorylation of ARNT tends to affect the AHR-ARNT interaction in some cells [77], [156], [150], [151], [159], [160]. CKII specifically phosphorylates ARNT isoform 1 in response to ligand-induced AHR nuclear translocation and is required for optimal AHR target gene regulation [77]. In addition, phosphorylation of HSP90 at S225 and S254 in the charged linker region within the cytosolic AHR complex weakens the HSP90-AHR protein interaction and may hinder ligand-activated transcriptional activation of the AHR [161].

Taken together, AHR translocation and DNA binding are regulated by phosphorylation of AHR and its interaction partners. Expression and activation of the involved kinases may vary between different cell types and in response to diverse stimuli and hence may contribute to the complexity of AHR activation.

### 9.2 SUMOylation

SUMOylation is the covalent attachment of the small ubiquitin-related modifier SUMO (a polypeptide) to a lysine residue (K) in the sequence ψKXE, where ψ is a large hydrophobic amino acid, X is any amino acid and E is glutamate [162], [163], [164], [165], [166]. The SUMO modification is a reversible process catalyzed by several enzymes including enzyme E1, conjugating enzyme E2, and ligase E3. DeSUMOylation is mediated by SUMO-specific proteases such as Sentrin-specific proteases 1–3 (SENP1-3) by cleaving the isopeptide bond at the lysine residue that removes the SUMO modification [167], [168], [169], [170], [171]. Several SUMO-specific proteases have been identified that exhibit different localization patterns, leading to differences in substrate specificity [170], [172], [173], [174], [175]. Several nuclear receptors and transcription factors are regulated by SUMOylation, e.g. GR [176], AR [177] or PPARG [178], affecting protein–protein interactions, activity, subcellular localization, and DNA binding [163], [179].

The first study on AHR and SUMOylation by Xing et al. showed that SUMOylation at K63 and K510 stabilized the protein by inhibiting its ubiquitination in MCF-7 cells [180]. SUMOylation repressed the transcriptional activity of AHR, and ligand treatment inhibited SUMOylation of AHR.

In addition to AHR, also ARNT and AHRR are regulated by SUMO-1 [181], [182]. SUMOylation of ARNT at K245 reduced its interaction with promyelocytic leukemia protein (PML), but had little effect on the transcriptional activity of AHR-ARNT [181]. Moreover, SUMOylation of AHRR at K542, K583 and K660 increased its transcriptional repressor activity and inhibited its interaction with the corepressors ANKRA2 (ankyrin repeat family A member 2), HDAC4 (histone deacetylase 4), and HDAC5 [182]. In addition, AHRR enhanced SUMOylation of ARNT and *vice versa*. Taken together, the SUMOylation status of AHR, ARNT and AHRR may contribute to the context-specificity of AHR activity.

Although the PTMs of AHR are not fully understood, there is increasing evidence that PTMs are involved in the regulation of AHR translocation, DNA binding and transcriptional activity. Some kinases, such as PKC and tyrosine kinases, are thought to be involved in AHR phosphorylation, but the involvement of other kinases remains nebulous. Furthermore, AHRR and ARNT PTMs have also been shown to affect AHR transcriptional activity. Context- and cell type-specific differences in the expression of PTM-mediating enzymes and substrates as well as cell type-specific effects of PTMs, may lead to differences in AHR activation and various context-specific biological responses. We refer to Table 1 for an overview of the PTMs of AHR and its interaction partners.

**Table 1:** PTMs of AHR and its interaction partners.

Protein	PTM	Site(s)	Involved Kinase/Enzyme	Effect of PTM	Cell type	Reference
AHR	phosphorylation	S12, S36	PKC	Impaired ligand-dependent nuclear translocation	COS-7 HeLa	PMID: 15063792 [144]
AHR	phosphorylation	S36	PKCθ	Enhanced nuclear import	T cells	PMID: 30214937 [145]
AHR	phosphorylation	S68	p38 MAPK	Inhibited nuclear export, nuclear accumulation	HaCaT	PMID: 14985336 [146]
AHR	SUMOylation	K63, K510	SUMO-1	Increased protein stability by preventing ubiquitination, Repressed AhR transcriptional activity	MCF-7	PMID: 22495806 [182]
HSP90	phosphorylation	K225, K254	n.d.	Weakens the functional AhR complex	Hepa1	PMID: 15581363 [161]
ARNT Isoform 1	phosphorylation	S77	CKII	Necessary for optimal AhR activity	Karpas 299, Peer	PMID: 35290121 [77]
ARNT	SUMOylation	K245	SUMO-1	Inhibited ARNT-PML protein interaction, thus slightly inhibited transactivation	MCF-7	PMID: 12354770 [162]
AHRR	SUMOylation	K542, K583, K660	SUMO-1	Increased transcriptional repression activity, necessary for efficient interaction with ANKRA2, HDAC4, and HDAC5	COS-7	PMID: 19251700 [163]

## 10 The role of epigenetics in AHR cell type-specificity

Each cell in the body has a unique repertoire of functions required for a particular tissue type or, more generally, in a specific context. These functions are enabled by specific gene expression programs, which are driven by accessibility of cell-specific regulatory regions in the chromatin [183]. Evolving single-cell technologies have shed light on such cell type-specific differences in open or closed chromatin [183], [184]. Chromatin accessibility is conferred by epigenetic modifications that orchestrate patterns of gene expression and shape functional differences between cell types and states. Three main classes of epigenetic mechanisms can be distinguished: DNA methylation, histone modifications, and non-coding RNA.

DNA methylation is a central epigenetic mark that occurs predominantly at positions in the DNA where a cytosine is followed by a guanine, at so called CpG sites. Methylation contributes to the silencing of gene expression directly by interfering with the binding of transcription factors, or indirectly by recruiting repressive methyl-binding proteins that recruit transcriptional co-repressors such as HDACs, leading to gene silencing [185], [186], [187]. Histones are the protein cores of the nucleosome that condense the DNA in the nucleus. Covalent modifications of the histone tails, e.g. methylation and acetylation, can convert chromatin structure into transcriptionally active or inactive regions and can recruit proteins involved in transcriptional regulation [187], [188]. HDACs for example are a group of enzymes that eliminate acetyl-groups from lysine residues of the histone tail leading to denser chromatin. Finally, non-coding RNAs are a large group of diverse untranslated RNA species that show a strong context-specific transcriptional pattern. Among others, they can alter the stability and translation of mRNA, and regulate recruitment of transcriptional regulatory complexes to chromatin [189].

Together these epigenetic modifications alter chromatin accessibility, thereby enhancing or reducing the transcriptional potential of the underlying DNA sequence and they determine and maintain gene expression programs that underlie cell fate [190], [191].

In the context of exposure to environmental chemicals, epigenetic silencing of either AHR itself or of its downstream targets involved in phase I metabolism or in anti-oxidative stress response, might lead to insufficient elimination of harmful chemicals or alterations in redox-balance and thereby contribute to disease development [192].

### 10.1 Epigenetic regulation of AHR

**Fig. 4:**
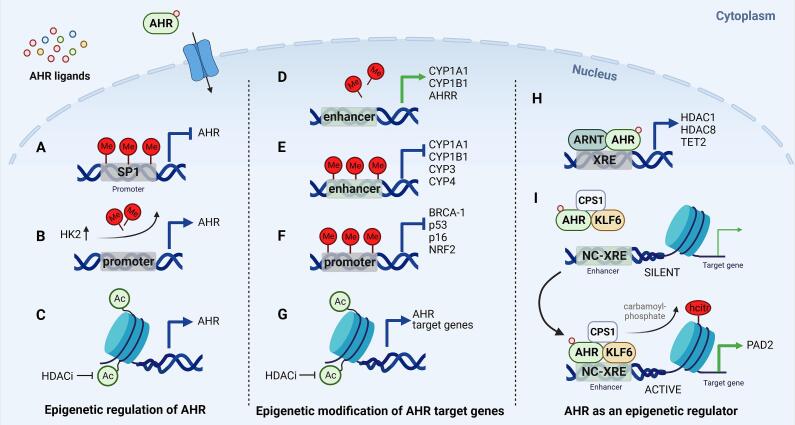
Epigenetic regulation of AHR signaling. AHR expression can be modulated by DNA methylation and histone modifications. (A) Hypermethylation of the AHR promoter impairs SP1 transcription factor binding and silences AHR transcription, e.g in acute lymphocytic leukemia. (B) Hexokinase 2 (HK2) is an AHR target gene that promotes AHR hypomethylation, which is associated with worse overall survival in several cancers. (C) Histone deacetylase inhibitors (HDACi), which prevent deacetylation and maintain an active chromatin state can induce AHR expression. (D) Enhancer hypomethylation and (E) hypermethylation transcriptionally regulate AHR target genes. (F) AHR-mediated promoter hypermethylation reduces expression of several tumor suppressors. (G) HDACi increase target genes expression in a variety of different cancer cell lines. (H) AHR target genes include the histone deacetylases HDAC1, HDAC8 and the DNA demethylating enzyme TET2 (Tet methylcytosine dioxygenase 2). (I) AHR-KLF6 (KLF transcription factor 6) binding to enhancer regions with non-canonical XREs (NC-XREs), e.g. binding site for AHR heterodimer without ARNT, leads to homocitrullination of H1K34 and induction of peptidyl arginine deiminase 2 (PAD2). Created with Biorender.com

Epigenetic modification of the *AHR* promoter has been described as a first regulatory level of *AHR* transcription. The proximal *AHR* promoter contains several binding motifs for transcriptional activators including SP1 binding sequences [193]. In acute lymphoblastic leukemia (ALL), hypermethylation of the AHR promoter was found in 33% of patients and impaired binding of SP1 resulting in *AHR* transcriptional silencing [194]. Suppression of *AHR* by hypermethylation has also been described for different cancer cell lines including the osteosarcoma cell lines U2OS and 143B, the diffuse large B-cell lymphoma cell lines U-2932 R2 and OCI-LY19, the chronic myeloid leukemia cell line K562 and the ALL cell lines Jurkat and REH [195], [196] (Fig. 4A). Recently, hexokinase 2 (HK2), an important enzyme for cancer growth, has been reported as an AHR target gene that modulates AHR activity by promoting *AHR* promoter demethylation [195]. Several cancers show negative correlation of *AHR* promoter methylation with *HK2* expression, which was associated with worse overall patient survival [195] (Fig. 4B).

Epigenetic regulation of *AHR* transcription is not only important in different cancers but has also been described for autoimmune disease. In rheumatoid arthritis, e.g. it has recently been shown that methylation of the CpG islands in the *AHR* promoter was significantly increased compared to controls [197].

Histone modifications also play a role in the regulation of *AHR* transcription*.* To date, the involvement of histone acetylation in expression of the *AHR* has been demonstrated in several cell lines. Constitutive expression of *AHR* was induced in murine Hepa-1 hepatoma cells by the HDAC inhibitors butyrate and trichostatin A (TSA) [193]. Also in human diffuse large B-cell lymphoma (U-2932 R2 and OCI-LY19), cervical carcinoma (HeLa), hepatoma (HepG2, Huh7), bronchial epithelial cells (16HBE), and in immortalized keratinocytes (HaCAT) TSA significantly induced AHR gene expression [194], [198], [199], [200] and it induced AHR-XRE luciferase activity in HepG2 [201]. However, in human MCF-7 breast cancer cells, TSA did not affect constitutive AHR expression levels [198] (Fig. 4C).

Conversely, in long-term estrogen-exposed MCF-7 cells increased *AHR* expression related to decreased histone 3 lysine 27 trimethylation (H3K27me3), which is generally associated with gene silencing and the maintenance of a repressive chromatin state [202].

Deregulation of micro-RNAs (miRNAs) as epigenetic and post-transcriptional regulators add another level of control to AHR expression and its downstream regulatory functions. Several miRNAs have been implicated in the control of AHR expression. MiR-124 inhibits AHR expression by directly targeting the 3′ untranslated region of AHR [203], [204], [205]. In colon tissues and intestinal epithelial cells of patients with Crohn’s disease miR-124 suppressed AHR on the protein level [204]. *AHR* also has been reported as a potential target of miR-375 based on studies in human HepG2 cells [206], which was further corroborated in mice [207]. In breast cancer cell lines (MCF-7 and MDA-MB-231) AHR was identified as a target of miR-548. Moreover, miR-122 was suggested to protect hepatocytes from acetaminophen toxicity among others by modulating AHR expression [208].

Taken together, these studies support a significant involvement of epigenetic mechanisms involved in the regulation of AHR expression that are highly dependent on the cell type.

### 10.2 Epigenetic modification of AHR target genes

#### 10.2.1 Regulation of AHR target gene expression by DNA methylation changes

More than three decades ago it was shown in nuclear extracts of murine hepatoma (Hepa1c1c7) cells that methylation in the *AHR* consensus sequence of the *Cyp1a1* enhancer inhibited AHR binding to this site [209]. Since then treatment with the DNA methyltransferase inhibitor 5-aza-2′-deoxycytidine (DAC) was reported to enhance basal expression of not only *CYP1A1* but also of *CYP1B1* in several human cell lines [198], [210], [211] (Fig. 4D).

Several human CYP-encoding genes have AHR consensus sequences in their 5′-regulatory regions, including *CYP1A1* and *CYP1B1* that are known to show tissue and cell type-dependent expression [212]. CpG islands covering the consensus sequences have been identified in the enhancer and promoter regions of these genes, suggesting DNA methylation as a modulating factor of their activity [209], [213], [214]. Indeed, previous studies have reported that P450 cytochromes can be regulated by DNA hypermethylation or hypomethylation in murine and human cell lines and also in certain cancerous tissues [185], [198], [210], [214], [215], [216]. In line, induction of *Cy1a1* expression by TCDD has been related to AHR-dependent demethylation at the *Cyp1a1* promoter [217]. Although, genes of the CYP2 and CYP3 family also harbor AHR-consensus sequences in their upstream regulatory regions they are generally weakly induced by AHR agonists. This observation has been linked to higher basal levels of DNA methylation compared to those found for *CYP1A1* and *CYP1B1* [218] (Fig. 4E).

Even in cells derived from the same tissue type, distinct DNA methylations in regulatory regions of CYP-encoding genes may occur. While pretreatment of HepG2 hepatoma cells with DAC increased expression of *CYP1B1* by the AHR agonist b-naphthoflavone (bNF), *CYP1B1* inducibility in HuH7 hepatoma cells was not affected by DAC pretreatment. This differential response was related to methylation at two CpG sites in XREs bound by AHR upstream of the *CYP1B1* promoter in HepG2 cells that was not observed in HuH7 cells [219]. In prostate cell lines, Okino et al. showed that DAC increased TCDD inducibility of *CYP1A1* in cancerous LNCaP cells but not in noncancerous PWR-1E and RWPE-1 prostate cell lines. In line, only LNCaP cells showed high methylation levels in the *CYP1A1* promoter, while their noncancerous counterparts did not. Also in human prostate cancer samples, the *CYP1A1* enhancer was methylated in 11 out of 30 samples, while no methylation in the *CYP1A1* promoter was detected in healthy control tissue [220] (Fig. 4E). Similar observations have been made for *Nrf2* (aka *Nlfe2l2*), another established AHR target gene and transcription factor that is known to crosstalk with AHR [221]. In murine models of prostate cancer, in LNCaP cells and in human prostate cancer tissue the *NRF2* promoter was hypermethylated and DAC treatment restored *Nrf2* transcription [222], [223]. NRF2 signaling is also highly context-specific and *NRF2* overexpression as well as repression have been associated with carcinogenesis and tumor aggressiveness in different cancer types [223]. Since NRF2 is a critical mediator in the antioxidant response, epigenetic changes affecting the interplay between AHR and Nrf2 signaling add to the complexity of its contribution to the development of adverse health effects, particularly those induced by environmental chemicals (Fig. 4F).

The AHR repressor *AHRR* is an important regulator of AHR and an AHR target gene [224]. Methylation changes at different CpG positions related to *AHRR* have been described in a multitude of studies investigating the effects of smoking and also particulate matter exposure based on DNA from blood samples [225]. Hypomethylation of *AHRR* associated to current or past smoking in adults, but it was also consistently shown in cord blood as a consequence of maternal smoking/environmental tobacco smoke exposure during pregnancy [226], [227]. AHRR hypomethylation has been associated with low lung function, increased lung cancer risk andmortality, and with smoking related morbidity and mortality [228], [229], [230], [231] (Fig. 4D). Epidemiological studies on effects of maternal smoking during pregnancy consistently report hypermethylation of CpGs located upstream of the *CYP1A1* promoter [232], [233], [234]. However, occupational exposure to PAH and current smoking was linked to hypomethylation in this region, which was suggested to contribute to increased oxidative stress, DNA damage and lung cancer risk [235], [236]. Interestingly, Tepkli et al., showed that in normal lung tissue *CYP1A1* was hypomethylated in current smokers, while in the cancerous tissue the same region was hypermethylated [236].

Although it is tempting to speculate that AHR activation by ligands present in tobacco smoke contributes to the methylation differences of *AHRR*- and *CYP1A1*-related CpGs, the opposing methylation differences suggest different underlying mechanisms that remain incompletely understood.

Epigenetic modifications not only affect classical AHR target genes. Also for several tumor suppressors AHR-mediated promoter hypermethylation has been described as a mechanism for transcriptional repression. TCDD enhanced promoter methylation of the tumor suppressors p16 (INK4a) and p53, thereby reducing their transcription [237]. Moreover, Papoutsis et al. observed AHR-dependent hypermethylation of the CpG island in the proximal promoter of *BRCA1* in MCF7 breast cancer cells [238] (Fig. 4F).

These findings, support the notion that unmethylated AHR consensus sequences are a prerequisite for AHR-DNA binding. However, it might not be sufficient as also other transcriptional coactivators such as p300 or transcription factors might be necessary and need to be expressed for a sufficient induction of AHR target genes [210], [216], [239].

Also other epigenetic modifications particularly histone modifications add an additional layer to AHR cell type-specific responses.

#### 10.2.2 Regulation of AHR target gene expression by histone modifications

The role of histone modifications in modulating AHR target genes has also mainly focused on the P450 enzyme coding gene family. Studies showed constitutive binding of HDAC1 to the *Cyp1a1* promoter contributing to its silencing [240], [241]. In line, inhibiting HDAC1, either by small molecule inhibitors or by siRNA, increased the constitutive expression of *CYP1A1* in human breast MCF-7 or cervical cancer Hela cells [198], in human HepG2 [201] and in murine Hepa1c1c7 liver carcinoma cells [240].

Moreover, AHR agonist activation was described to alter histone modifications in the *CYP1A1* promoter. Ovensen et al. found that different xenobiotic ligands of the AHR induced similar histone modification patterns, including an increase in H3K4me3 and H4K16Ac in the *Cyp1a1* promoter of Hepa-1 cells [242]. Also, in mouse primary hepatocytes, TCDD treatment enhanced H3K4me3, and H4Ac in the *Cyp1a1* promoter with a concomitant decrease in the repressive marker, H4K20me3 [217].

In MCF7 cells, TCDD led to an induction of H3K4me3, H4Ac, H3K9Ac, H3K14Ac in the *CYP1A1* and *CYP1B1* promoters [210]. While similar histone modifications were found in the *CYP1A1* promoter of HepG2 cells, the promoter region of *CYP1B1* showed less activated histone marks compared to MCF-7 cells, both before and after TCDD treatment, consistent with a lack of inducibility of *CYP1B1* in HepG2 cells [210]. On the other hand, in prostate cancer LNCaP cells that lack H3K4me3 in the *CYP1A1* regulatory region, TCDD did not induce *CYP1A1* expression.

The HDAC inhibitors butyrate, panobinostat and vorinostat enhanced TCDD-mediated induction of *Cyp1a1/CYP1A1* in the murine YAMC and human Caco-2 colon carcinoma cell lines, respectively. While *CYP1B1*, *AHRR* and *TIPARP* were enhanced by TCDD in Caco-2 cells, they were only marginally induced in YAMC cells [243]. Butyrate treatment of Caco-2 cells led to recruitment of AHR and the polymerase II to the *CYP1A1* promoter and enhanced H3K47Ac and H3K9Ac there [243]. Further experiments underline the cell type specificity of histone modifications in the expression of CYP encoding genes. In the neuroblastoma cell lines UKF-NB-3 and UKF-NB-4 TSA and valproic acid, decreased the mRNA expression of *CYP1B1* [244], while these inhibitors have been shown to activate *CYP1A2* expression in Hep3B, MCF-7 and in Hela cells [198], [245].

Altogether, these studies reveal the importance of histone modifications in the expression of AHR target genes and underline the cell type specificity effects related to histone modifications.

#### 10.2.3 Regulation of AHR downstream effects by micro-RNAs, and long non-coding RNAs

AHR regulates several non-coding RNAs that may affect AHR-mediated downstream signaling and function in different species [246], [247].

Sex-determining region Y-related (SRY) high-mobility group box 4 (SOX4) is known to promote tumorigenesis and is highly expressed in different tumor types [248]. Decreased expression of *SOX4* has been attributed to induction of miR-335 and the miR-213/132 cluster via activation of AHR. Different AHR-activating ligands including TCDD, 6-methyl-1,3,8-trichlorodibenzofuran (MCDF), gallic acid (GA) and Flavipin induced miR-335 and miR-213/132 thereby decreasing SOX4 expression [249], [250], [251]. AHR inhibition prevented these ligand-mediated effects of miR-335, miR-213/132 on *SOX4* expression confirming AHR interaction with these miRNAs [250], [251]. Agonist activation was associated with decreased migration and invasion of breast cancer cells and inhibited lung metastasis in a mouse model. SOX4 overexpression partially blocked TCDD and MCDF-mediated inhibition of breast cancer cell (MDA-MB-231) migration and invasion in line with the oncogenic role of SOX4. These findings suggest, that the antitumor effects of AHR-miR-213/132/miR-335 axis activation might depend on SOX4-baseline expression levels [250]. On the other hand, in multiple melanoma, activation of the AHR was implicated in inactivation of the tumor suppressor gene p53. Several miRNAs have been reported to target p53 in multiple melanoma cells, including miR-25. Direct binding of AHR to miR-25 was confirmed after exposure of multiple melanoma MM1.S cells to BaP and TCDD, with a concomitant upregulation of miR-25 [252].

The role of AHR in the differentiation of T cell subpopulations such as IL-17-expressing T helper cells (Th17) and regulatory T cells (Treg) and its significance for autoimmune disease has been established [51], [52], [253]. Studies exist that implicate AHR-dependent miRNA expression in this process. The microRNA miR-132/212 cluster was shown to be upregulated in an AHR-dependent manner [251] and to promote Th17 cell differentiation [254], [255]. A role of the AHR/miR-132/212 axis has been established in murine models of colitis and of experimental autoimmune encephalomyelitis [254], [255].

Further miRNAs have been implicated in AHR-mediated differentiation of Th17 and Treg cells in a ligand-dependent fashion. Indole-3-carbinol (I3C) and 3,3′-diindolylmethane (DIM) increased *Il17*-targeting miR-495 and miR-1192 while decreasing miRNA-31, miRNA-219 and miRNA-490 that target *Foxp3* only in AHR^+/+^ wild type mice. The opposing effect on this miRNA profile was observed when using FICZ. In line, I3C and DIM attenuated Th17 generation and promoted Treg while the opposite was observed for FICZ [256].

Taken together, these studies corroborate a role of AHR-mediated miRNAs expression that can differ in a ligand- and cell type-dependent fashion.

### 10.3 AHR as an epigenetic regulator

AHR might function as an epigenetic regulator by altering the chromatin structure through its direct interaction with transcriptional co-activators or repressors and by mediating histone- or DNA-modifying enzymes.

Several reports exist that show AHR involvement in regulation of chromatin accessibility and remodeling. AHR can interact with transcriptional cofactors such as the mediator complex [257], GRIP1-associated coactivator 63 (GAC63) [258] or with the chromatin remodeling complexes SWI/SNF [259] and nucleosome remodeling and deacetylase (NuRD) [260]. These interactions with the transcriptional machinery influence basal gene expression but also transcriptional induction and repression of AHR target genes [257], [258], [261]. As there is a general plasticity in the composition of transcription pre-initiation complexes, differences in interaction of AHR with the transcriptional machinery might contribute to the context-specific response of AHR.

As indicated above, AHR may also function as an epigenetic regulator by modulating expression of histone- and DNA-modifying enzymes.

HDAC8 has been reported as an AHR target as it was induced by TCDD and by overexpression of AHR in hepatoma cell lines. Also in hepatocellular carcinoma patients, HDAC8 expression significantly correlated with AHR transcription and protein levels. As HDAC8 inhibition had an anti-proliferative effect in hepatoma cells, it has been suggested that xenobiotics can alter the epigenetic dynamics by stimulating the AHR-HDAC8 axis and thereby contributing to hepatocarcinogenesis [262]. In contrast, AHR-mediated upregulation of HDAC1 and retinoblastoma 2 (Rb2) by 3-methylcholanthrene (3MC) that decreased cell proliferation in vascular endothelial cells [263]. However, high levels of HDAC1 have been found in different tumor types and have been associated with poor survival [264]. While knockdown of HDAC1 in cancer cells impairs G2/M transition and inhibits cell growth, its expression induces p53 deacetylation, repressing p53-mediated apoptosis [265]. Therefore, ligand mediated AHR activation might contribute to the tumor promoting effects associated with HDAC1 (Fig. 4H).

AHR was also shown to directly promote histone modification. Ligand activation led to binding of an AHR-KLF6 complex to enhancer regions in the DNA [266]. This triggered the recruitment of carbamoyl phosphate synthase 1 (CPS1), resulting in homocitrullination (aka carbamylation) of H1K34 which in turn promoted chromatin activation and upregulation of the peptidyl arginine deiminase 2 (PAD2) [266]. PAD enzymes convert arginine to citrulline leading to a PTM called citrullination. Thus, recruitment of CPS1 to the AHR-KLF6 heterodimer might promote carbamylation and citrullination of intracellular proteins. The role of this pathway in cancer has so far not been further investigated, however carbamylated proteins play a role in the pathophysiology of autoimmune diseases such as rheumatoid arthritis [267] (Fig. 4I).

Ten-eleven translocation enzymes (TETs) are dioxygenases that catalyze the oxidation of methylated cytosines in the DNA to promote demethylation. Chen et al. demonstrated that the *TET2* promoter contains an AHR-binding site, which the receptor binds after its activation with Kyn. TET2 induction by AHR ligand activation promotes demethylation at the promoter of the 5′-nucleotidase *NT5E*, which encodes the membrane-bound CD73 protein that converts AMP to the anti-inflammatory adenosine. In SLE, downregulation of AHR was associated with increased methylation levels at the *NT5E* gene promoter, suggesting a contribution to reduced adenosine production in Treg and likely B cells of SLE patients [268], [269] (Fig. 4H).

In summary, AHR may affect histones modifications through transcriptional regulation of HDACs, by recruitment of histone modifiers and can promote DNA demethylation by inducing TET2 expression. Although studies showing direct involvement of the AHR in the regulation of the expression of epigenetic modifiers are sparse, reports so far suggest that this avenue of AHR activation should further be explored.

## 11 Expression of different factors that degrade AHR ligands

Activation of the AHR induces CYP enzymes which in turn modify AHR ligands, leading to their metabolic clearance [87], [270], [271], [272]. Hence, an autoregulatory feedback loop exists between the AHR and the AHR-induced CYP enzymes that control the duration and intensity of AHR activation. In cell culture, overexpression of CYP1A1, 1A2, or 1B1 reduced AHR-mediated luciferase reporter activity, while a specific inhibitor of CYP1B1 partially blocked the CYP1B1-mediated reduction in reporter gene activity [270]. Soluble extracts from Ahr^−/−^ mouse lung tissue induced a 4-fold increase in AHR reporter activity compared to extracts from wild type lungs, suggesting that in the absence of the Ahr less AHR ligands are degraded [270]. Also *in vivo* in mice, constitutive expression of *Cyp1a1* diminished the levels of endogenous AHR ligands resulting in an almost AHR-deficient state, which was rescued by increasing the intake of AHR ligands [271].

This feedback mechanism implies that the levels and the activity of the CYP enzymes can affect AHR activity. Indeed, Wincent and colleagues demonstrated that multiple factors that inhibit CYP activity including UVB irradiation, hydrogen peroxide (H_2_O_2_), the photochemical crosslinker trioxalen, the antifungal agent ketoconazole, the DNA intercalator ellipticine, the dietary supplement diosmin as well as the protein synthesis inhibitor cycloheximide activate the AHR by inhibiting the degradation of trace amounts of FICZ contained in cell culture medium [272]. Compounds inhibiting the CYPs could hence be mistakenly regarded as AHR agonists because the concentration of endogenous agonists and subsequent AHR activity would increase. Diverse endogenous compounds including arachidonic acid and porphyrins, which have been implicated in activating the AHR, are also substrate of CYPs [273], [274]. It remains to be comprehensively determined which endogenous AHR agonists are degraded by the CYP enzymes and accumulate upon CYP inhibition.

Moreover, any alteration in CYP expression or activity may affect AHR activity. Multiple factors including epigenetic mechanisms such as changes in DNA methylation [185], [198], [209], [210], [213], [214], [215], [216] as well as other transcription factors such as PPARA, constitutive androstane receptor and retinoic acid receptor alpha can modulate the constitutive and inducible expression and activity of the CYP enzymes, possibly resulting in highly context-specific degradation of AHR ligands [273], [275]. Also CYP1A1 dietary competitive substrates can lead to enhanced systemic AHR ligand distribution from the gut, increasing AHR activation in key barrier tissues [276]. Modulation of CYP enzyme activity may hence contribute to the complex biology of AHR activity in cancer and beyond.

The AHR antagonist 3′-methoxy-4′-nitroflavone also inhibited FICZ degradation and led to a delayed increase in CYP1A1 induction [272]. As AHR inhibition generally reduces CYP enzyme levels, AHR inhibitor treatment likely leads to an accumulation of endogenous ligands that can activate the AHR and lead to rebound activation once the levels of AHR inhibitor decrease. This phenomenon may require particular attention concerning the dosing of AHR inhibitors.

## 12 Conclusion

The AHR is a highly complex and versatile protein that exhibits a wide range of functional aspects. Its complexity arises from the diversity of its ligands, expression levels, interactions with co-regulatory proteins, crosstalk with other factors, non-transcriptional effects, epigenetic regulation, and posttranslational modifications. Understanding the intricate interplay of these factors is essential for unraveling the diverse functions of AHR and its implications in environmental responses, cellular homeostasis, and disease pathogenesis. While the complexity of AHR signaling may appear daunting for interventions such as cancer therapy, the AHR shares such complexity with many other drug targets. The main question hence is how to best tackle this complexity in order to move forward. Rather than investigating tumors based on historically evolved characteristics including their tissue of origin or established molecular markers, we propose to identify subgroups of cancers, in which AHR mediates similar functions. These shared functions may indicate common regulation of AHR activity. In depth analysis of these subgroups will shed light on whether similar functions indeed can be used as surrogates for common regulation and whether interventions in patients belonging to such an AHR function subgroup will yield similar clinical effects.

Continued research in this field will deepen our knowledge of AHR biology and shed light on its therapeutic potential in cancer and beyond.

## CRediT authorship contribution statement

*Christiane A. Opitz:* Conceptualization, Writing – original draft, Writing – review & editing. *Pauline Holfelder:* Writing – original draft, Writing – review & editing, visualization. *Mirja Tamara Prentzell:* Writing – original draft, Writing – review & editing. *Saskia Trump:* Conceptualization, Writing – original draft, Writing – review & editing, Visualization.

## Declaration of Competing Interest

ST and CO are founders and CO is managing director of cAHRmeleon Bioscience GmbH. Authors of this manuscript have patents on AHR inhibitors in cancer (WO2013034685, CO); A method to multiplex tryptophan and its metabolites (WO2017072368, CO); A transcriptional signature to determine AHR activity (WO2020201825, ST, CO); Interleukin-4-induced gene 1 (IL4I1) as a biomarker (WO2020208190, ST, MTP, CO); Interleukin-4-induced gene 1 (IL4I1) and its metabolites as biomarkers for cancer (WO2021116357, ST, CO).
